# ANRIL: Molecular Mechanisms and Implications in Human Health

**DOI:** 10.3390/ijms14011278

**Published:** 2013-01-10

**Authors:** Ada Congrains, Kei Kamide, Mitsuru Ohishi, Hiromi Rakugi

**Affiliations:** Department of Geriatric Medicine and Nephrology, Osaka University Graduate School of Medicine, 2-2 B6, Yamadaoka, Suita, Osaka 565-0871, Japan; E-Mails: ada@geriat.med.osaka-u.ac.jp (A.C.); ohishi@geriat.med.osaka-u.ac.jp (M.O.); rakugi@geriat.med.osaka-u.ac.jp (H.R.)

**Keywords:** ANRIL, CDKN2BAS, polycomb proteins, 9p21

## Abstract

ANRIL is a recently discovered long non-coding RNA encoded in the chromosome 9p21 region. This locus is a hotspot for disease-associated polymorphisms, and it has been consistently associated with cardiovascular disease, and more recently with several cancers, diabetes, glaucoma, endometriosis among other conditions. ANRIL has been shown to regulate its neighbor tumor suppressors CDKN2A/B by epigenetic mechanisms and thereby regulate cell proliferation and senescence. However, the clear role of ANRIL in the pathogenesis of these conditions is yet to be understood. Here, we review the recent findings on ANRIL molecular characterization and function, with a particular focus on its implications in human disease.

## 1. Introduction

### 1.1. The Chr9p21 Locus

Recent findings from the genome-wide association studies (GWAS) have been pointing to novel disease-associated DNA regions, opening doors for new and more accurate targets for research and future therapies for several diseases. One of these new loci is the chromosome 9p21, which was highlighted as the strongest genetic susceptibility locus for cardiovascular disease (CVD) [1,2] and later linked to other conditions such as type 2 diabetes [2,3], Alzheimer disease [4], glaucoma [5,6], endometriosis [7] and periodontitis [8]. Interestingly, the CVD and diabetes-associated hot-spot lies in a region previously considered a gene desert, and overlaps the sequence of a newly discovered long non-coding RNA (lncRNA), ANRIL (antisense non coding RNA in the *INK4* locus). Remarkably, variants in this region have also been associated with several cancers: leukemia [9], breast cancer [10], basal cell carcinoma [11], melanoma [12,13], pancreatic carcinoma [14], ovarian cancer [15], and glioma [16].

In the vicinity of the disease-associated haplotype block, three genes, *CDKN2A*, *CDKN2B*, and methyl-thioadenosine phosphorylase (*MTAP*) appear as potential candidates to mediate the associations. *CDKN2A* encodes for two distinct proteins, p16 and ARF, using alternative reading frames. CDKN2A/ARF and CDKN2B are known tumor suppressors and have well-established roles in cell proliferation, apoptosis, senescence and aging [17,18]. Deletion and inactivation of *MTAP* have also been connected to cancerogenesis [19,20]. It seemed plausible that the disease-associated SNPs would be regulating the expression of these genes. However, most of the disease-associated polymorphisms have been associated with the expression of ANRIL rather than *CDKN2A/B* or *MTAP* in several reports [21–23].

The 9p21 association with cardiovascular disease (CVD) presents an additional appeal besides being the strongest genetic factor identified by the genome wide association studies (GWAS). It is independent from most traditional risk factors [2], and therefore, it is likely to represent a component of the disease hitherto unknown and probably not targeted by current treatments.

Many of the polymorphisms in the 9p21 locus disrupt several predicted transcription factor binding sites [24] suggesting that the locus expression is regulated by numerous signaling pathways. Notably, some of these transcription factors are involved in key physiological processes such as inflammation, RAS response, and FOXO-Sirtuin-longevity signaling. ANRIL mediates the response to at least two of these signaling pathways: STAT1 [24] and RAS [25]. In addition, different disease-associated SNPs in the region have proven to modulate independently the expression of ANRIL and *CDKN2A/B*. In particular, the CVD-associated polymorphisms have been shown to regulate ANRIL expression *in vitro* [24] and also in human cohorts [21–23]. Seemingly, the polymorphisms act through independent mechanisms and some of them, at least, seem to be mediated by ANRIL.

### 1.2. The Long Non Coding RNA in the Chromosome 9p21: ANRIL

ANRIL is a long non-coding RNA (lncRNA) transcribed by RNA polymerase II [26], and spliced into multiple linear isoforms [27,28] including an ANRIL-MTAP fusion transcript [29]. Most of the splicing variants are polyadenylated; however, some circular non-polyadenylated variants have also been described [29]. Some of the splicing variants have been reported to be tissue-specific [28,29], suggesting their physiological relevance and underlining the complexity of its regulatory function.

ANRIL exons expression is detectable by RT-PCR, but their expression is low, as it is the case of other functional non-coding RNAs. Despite the numerous splicing variants that have been described, the characterization of the isoforms in different cell lines and tissues is still incomplete. However, the importance of the discrete long isoforms needs to be clarified; it is possible that the functionality of this non-coding RNA do not rely on the complete transcripts. In fact, analysis of next-generation RNA-sequencing results revealed a disparity in abundance between ANRIL exons [29]. Moreover, short non-coding RNAs forming hair-pin structures are already shown to be expressed from Polycomb-repressed loci and have a role in their repression in *cis* [30]. There seem not to be a simple motif or conserved structure in long non coding RNAs (lncRNAs) that is universally recognized by Polycomb proteins; however, there is evidence of structural patterns [31,32] and, in some cases stem-loop structures that preferentially bind these chromatin modifying proteins. An example of the requirement of such a structure is RepA which contains a conserved stem-loop structure generated by Xist transcript which is sufficient to recruit PRC2 *in vivo* [33,34]. Partial ANRIL transcripts carrying these structural patterns could be acting as independent regulatory molecules, rather than one or few long transcripts.

Interestingly, the central exons (Exon 4 to 12) of ANRIL seem to be the least abundant [29]. Those middle exons form rare circular isoforms whose expression is associated with the CVD-risk alleles [29]. Although the function of these circular variants of ANRIL is yet to be clarified, there is already evidence suggesting the importance of circular antisense transcripts [35].

## 2. ANRIL, Polycomb Proteins and Epigenetic Regulation of the CDKN2A/B Locus

Polycomb proteins are a group of proteins involved in transcriptional gene repression via two distinct histone modifications: trimethylation of lysine 27 on Histone 3 (K27H3) and monoubiquitination of histone H2A. They are key developmental regulators that play critical roles in differentiation, maintenance of cell identity [36] and cancerogenesis [37,38]. There have been several reports focused on their role on cancer development and progression; however, there are indications that the gene expression changes driven by histone modifying proteins control several aspects of human health [39–41].

Polycomb proteins work in multi-protein complexes, the Polycomb repressive complex 1 (PRC1) and 2 (PRC2). PRC2 is involved in the initiation of silencing by catalyzing the methylation of lysine 27 on Histone 3 (K27H3). PRC2 contains three subunits EED, SUZ12 and the methyltransferase EZH1/2. The PRC1 complex is involved in the maintenance of silencing of the target genes. PRC1 comprises BMI1, mPh1/2, a Chromobox (CBX) and the RINGA/B, a ring finger protein with ubiquitin E3 ligase activity [42]. Several chromobox proteins have been described and each with a distinct pattern of binding in the chromatin [43], suggesting that the CBX unit confers specificity to the complex. Particularly, CBX7 has an important role in cellular life span [44] and is directly involved in the regulation of several genes frequently silenced in cancer [37]. Polycomb repressive complexes are critical in the epigenetic regulation of the CDKN2A/B locus (reviewed in reference [45]). Moreover, histone modifications caused by Polycomb proteins occur in coordination with other epigenetic mechanisms. Polycomb proteins CBX7 and EZH2 interact with DNA methyltransferase DNMT3B and the activity of chromatin remodelers have shown to influence PRCs occupancy in the CDKN2A/B locus [45].

The association between long non-coding RNAs (lncRNAs) and polycomb (Pc) proteins to induce silencing is emerging as a common mechanism of epigenetic regulation. Other well documented examples of this kind of interaction are: Hotair [46], Kcnq1ot1 [47] and RepA [34], which have been reported to associate with polycomb proteins, acting as cofactors necessary for the polycomb proteins to silence their target loci. Growing evidence suggests that a large proportion of lncRNAs bind chromatin modifying proteins to alter expression patterns in different cell types [48].

ANRIL has been shown to specifically bind two polycomb proteins: CBX7 (PRC1) and SUZ12 (PRC2) [25,26,42], to regulate histone modification in the *CDKN2A/B* locus. Competitive inhibition of ANRIL transcript by expression of an antisense sequence impairs CBX7-mediated repression of the *CDKN2A* locus and causes a concomitant shortening of cellular life span [26] in human fibroblasts. Yap *et al.* also identified several RNA loop structures formed by ANRIL transcript, which specifically bind CBX7, and at least one of them participate in CBX7 recognition of histone methylated lysine (H3K27). CBX7 recognition of H3K27 is required for the mono-ubiquitination of Histone 2A in lysine 119 (H2A-K119) which in turn results in the maintenance of repression in the locus [26]. Intriguingly, ANRIL depletion caused the up-regulation of *CDKN2B* but not *CDKN2A/ARF* in another fetal fibroblast cell line [25]. This study demonstrated that ANRIL binds SUZ12 (component of Polycomb Repressive Complex 2) and influence cellular proliferation via regulation of *CDKN2B*.

The over-expression of an ANRIL splicing variant in Hela, causes the down-regulation of several genes involved in important chromatin architecture remodeling mechanisms [49]. Remarkably, one of the most down-regulated genes was co-activator p300 (also known as *EP300*) which is a transcriptional co-factor with histone acetyl-transferase activity [50]. P300 has numerous gene targets and localizes at enhancer sequences where it is involved in cell-type-specific gene expression determination [51]. Co-activator P300 has histone acetyltransferase activity, activates several loci related with senescence [52] and its patterns of occupancy change with aging [53]. Other genes suppressed by ANRIL over-expression have been previously linked to histone modification [54–56].

The deletion of the chromosome 9p21 orthologous region in mice caused a severe reduction of expression of *CDKN2A/B*, particularly in aortic and cardiac tissues [57]. This is consistent with the presence of multiple regulatory sequences identified in the CAD risk interval [24]. The deleted orthologous interval encompasses the region that in humans encodes for the last exons of ANRIL [58]. The mice 70 kb interval, orthologous to the human 58kb coronary artery disease (CAD) risk region contains part of an antisense non-coding RNA, AK148321, as well as other annotated transcripts; however, there is no homology between ANRIL and the antisense transcripts identified in mice. Moreover, the effect of the deletion reported by Visel *et al.* seems inconsistent with the inhibitory effect of ANRIL upon CDKN2A and CDKN2B reported by several groups [25,26]. ANRIL has been proposed to be required for the recruitment of polycomb proteins and consequent repression of the *CDKN2A/B* locus. In this sense, a similar role of the antisense transcript in mice seems unlikely. It seems more plausible that the reduced expression of *CDKN2A/B* observed by Visel *et al.* comes as a result of the deletion of important regulatory sequences rather than the regulatory effect of the deleted exons of the AK148321 transcript. Furthermore, the regulation of the locus during senescence and oncogenic stimuli is intrinsically different in human and mice [59,60], which difficult to extrapolate these results into the human locus. Further study is required to investigate the role of this transcript in the regulation of the *CDKN2A/B* locus in mice.

More interestingly, the targeted deletion of the orthologous CAD-risk interval effect upon *CDKN2A/B* in mice proved to be allele specific [57], suggesting that the action of the human transcript, ANRIL, could depend on its tethering to the chromosome where it is encoded. However, there is experimental evidence demonstrating ANRIL is able to silence *CDKN2B* promoter in *trans*, although less effectively than in *cis* [61].

Long non-coding RNAs are able to repress and activate gene expression via several mechanisms, and both, *cis-* and *trans-*acting long non-coding RNAs have been reported [62]. ANRIL has a soundly established role recruiting polycomb proteins in *cis*. However, the fact that ANRIL over-expression is able to induce histone modification in a distant CDKN2B promoter (acting in *trans*) [61], suggests that the local transcription of ANRIL is not indispensible for at least some of its regulatory function. The tantalizing possibility of a trans-acting ANRIL also highlights the necessity of comprehensive genome-wide approaches to unveil possible distant targets of this non-coding RNA.

Remarkably, ANRIL over-expression also induced DNA hyper-methylation of the locus in differentiated cells [61]. Although, evidence suggests a common role of non-coding RNAs precluding DNA methylation of their neighboring CpG islands, non-coding transcripts originated in the promoter of ribosomal RNA genes represent an example of non-coding RNAs inducing hyper-methylation in *cis* [62]. Furthermore, CBX7 is known to complex with DNA methyltransferase enzymes and therefore participates in DNA methylation of several loci involved in cancer [37], raising the question whether CBX7-ANRIL complex might also be mediating the effect on DNA methylation reported by Yu *et al.* [61].

ANRIL over-expression caused predominantly suppression of gene expression in HELA [49], and this is compatible with ANRIL being required by polycomb proteins to repress gene expression. However, there were some few genes up-regulated by ANRIL over-expression in this study. The gene up-regulated the most was *RREB1* (Ras responsive element binding protein) [49], a transcription factor that binds to RAS-responsive elements of gene promoters. Remarkably, there is a predicted binding site for RREB1 in the 9p21 locus, which is disrupted by one of the disease-associated SNPs (rs564398). Moreover, ANRIL expression is inhibited by RAS [25], possibly through RREB1. It seems plausible that RAS induces RREB1 activity to bind the responsive element within ANRIL locus and therefore inhibit its expression; ANRIL depletion, in turn, impairs repression of *CDKN2B* with the consequent up-regulation of *CDKN2B*. These connections suggest a regulatory feedback between ANRIL and the RAS signaling pathway, strongly associated with cancerogenesis. This is a single example of the complexity of the regulatory landscape of the 9p21 locus and the possible implication of ANRIL in mediating the associations detected in the region. Furthermore, the existence of genes up- and down-regulated by ANRIL [49,63] that do not seem downstream to *CDKN2A/B* is suggesting a wider regulatory panorama for this non-coding RNA.

## 3. ANRIL Expression in Disease

ANRIL is over-expressed in leukemia patients leukocytes compared with normal controls, while *CDKN2B* showed the opposite pattern of expression [61]. Remarkably, GWASs have detected a risk allele for acute lymphoblastic leukemia in the first exon of *CDKN2A* [9], coinciding with the promoter region of ANRIL. ANRIL is also up-regulated in prostate cancer tissues in comparison with normal epithelial cells, accompanied by down-regulation of *CDKN2A* [26].

*In vitro*, ANRIL depletion has been associated with reduced proliferation, suggesting a pro-cancerogenic role of the transcript. This view is also supported by the up-regulation of ANRIL observed in leukemia [9], prostate cancer tissues [26] and in the risk allele carriers for basal cell carcinoma (BCC) and glioma [23]. However, the T allele of the polymorphism rs2151280 was correlated with an increased number of plexiform neurofibromas and also with reduced ANRIL expression in patients suffering from a tumor predisposition syndrome [64]. In addition, a melanoma associated variant rs1011970-T was also associated with the reduction of ANRIL expression [23]. ANRIL exons show different expression levels and these studies target different regions of ANRIL for the expression quantification, complicating the interpretation of these correlations, see Table 1.

Pro-oncogenic RAS inhibits ANRIL expression and activates *CDKN2B* [25]. Interestingly, one of the SNPs most strongly correlated with ANRIL expression, rs564398, [21,23] is predicted to disrupt “Ras responsive element binding protein 1” (RREB1) binding site in the 9p21 locus [24]. RAS oncogenes have a well-documented role in cancer pathophysiology [67], but RAS also participates in atherosclerosis progression, promoting vascular senescence and inducing pro-inflammatory cytokines expression [68].

Additionally, the silencing of ANRIL reduces proliferation in fibroblasts [26] and vascular smooth muscle cells [21]. This reduced proliferation might be the result of premature senescence, an important mechanism to prevent tumorogenesis but also implicated in endothelial dysfunction [69] and inducing a pro-inflammatory phenotype in vascular smooth muscle cell [68].

The presence of multiple enhancers [24] and CCCTC-binding factor (CTCF) binding sites [24,70] in the 9p21 region suggests that its transcripts, CDKN2A/B/ANRIL, are subject to complex temporal and tissue-specific regulation. The cell-type specific effect of the transcription factor STAT1 upon ANRIL expression represents an illustration of the complex regulatory panorama underlying the chr9p21 associations. The pro-inflammatory cytokine, Interferon gamma, stimulates STAT1 activity and binding to a site harboring two risk variants for CAD in the 9p21 locus. The binding of STAT1 induces the expression of ANRIL, and represses CDKN2B in endothelial cells. However, the silencing of STAT1 in the non-risk-haplotype-carrier Lymphoblastoid cells (LCLs) induces expression of ANRIL, suggesting that the binding of STAT1 reduces expression of ANRIL in LCLs [24].

ANRIL expression was also up-regulated in gingival epithelial cells and gingival fibroblasts during bacterial infection, which supports the role of ANRIL in inflammatory response [8]. In this study the increased expression of ANRIL was accompanied by reduction in CDKN2A expression, and no effect on CDKN2B expression.

Cardiovascular disease (CVD) risk-alleles in the 9p21 region have been associated with ANRIL expression. However, the CVD-risk alleles have been associated with both, increased [22] and reduced expression of ANRIL [21,23]. Holdt *et al.* presented a meticulous analysis of 4 CVD-associated SNPs and their role in the expression of some of ANRIL splicing variants and CDKN2A/B in a large patient sample. In this study, they elegantly associated an increased expression of ANRIL with the risk alleles and atherosclerosis severity. However, the analysis of two other smaller volunteer samples: 487 [23] and 57 [21] individuals, evaluating different exons, correlated the risk alleles with a reduction of ANRIL expression. Diabetes-associated risk variants (rs10811661-T and rs2383208-A) were associated with a down-regulation of ANRIL expression [23].

This locus has been focus of particular interest throughout the last decades because of its connection with cancer [71], aging [17,72] and more recently its role in stem cell reprograming [73]. However, there are some unclear points in the role of CDKN2A/B in human health and aging. Although, CDKN2AB/ARF locus overexpression seems to have a positive over-all effect in life span in mice [17], there is some contradictory evidence regarding the role of these tumor suppressors in disease. CDKN2A/B locus is silenced in young healthy cells and its expression is stimulated by age and oncogenic insults [71]. This increase in expression (particularly of CDKN2A) is associated with the impaired regenerative potential of several tissues observed during aging [74,75]; however, CDKN2A/B is silenced or deleted in a wide-range of human cancers [71], suggesting it has a key role preventing cancerogenesis. Certainly, the regulation of this locus may be at a key cross point between aging, healthy regenerative proliferation and tumorogenesis. The idea that this non-coding RNA might shed new light on the mechanisms regulating this locus is enticing.

## 4. Conclusions and Discussion

Non-coding RNAs are long known to mediate gene expression regulation by a variety of mechanisms [76]. The literature is dominated by reports of small non-coding RNAs, such as microRNAs; however, long non-coding RNAs are arising as important regulators of gene expression. The long non-coding RNA in INK4 locus, ANRIL, is encoded in a genetic region highlighted for its connection with several human diseases and the material reviewed herein suggests that ANRIL has an important role in mediating the associations detected in the 9p21 locus.

**Figure 1:**
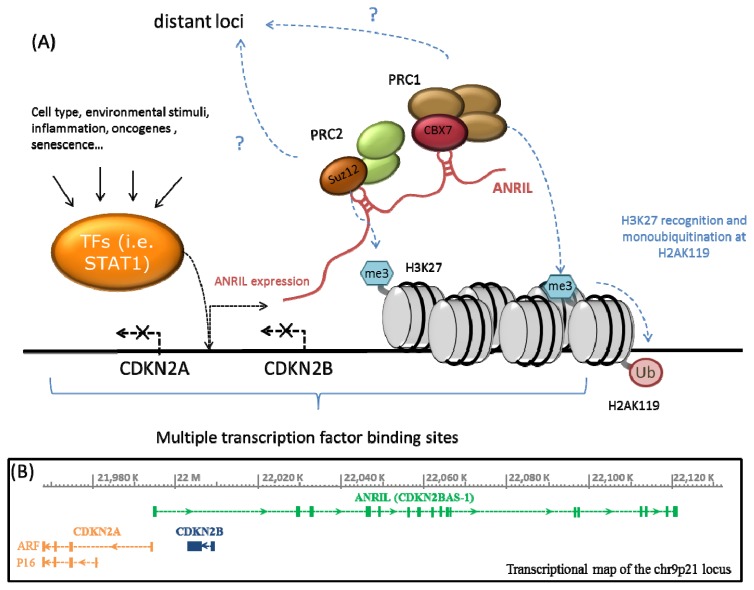
(**A**) Chr9p21 region is rich in regulatory elements and several transcription factor (TF) binding sites have been predicted along its sequence. These transcription factors are activated in response to external factors and signaling pathways, in a cell-type specific manner. TF binding possibly activates or represses the expression of ANRIL. Polycomb protein complexes 1 and 2 (PRC1 and PRC2), have RNA binding domains in their subunits CBX7 and SUZ12 respectively. ANRIL binds SUZ12 subunit of PRC2 to induce methylation of histone 3 in the lysine 27 (H3K27) and consequent silencing of the CDKN2A/B locus. ANRIL binds CBX7 in PRC1 which allows the recognition of H3K27 necessary for the monoubiquitination at histone 2A at lysine 119 (H2AK119) and maintenance of silencing. Therefore, ANRIL modulation impacts in the repressing ability of Polycomb proteins, inducing or inhibiting expression of CDKN2A/B and possibly other distant loci by histone modification. The disease-associated alleles might impair TF binding and response to different stimuli, alter ANRIL/CDKN2A/B expression (and possibly other loci) and contribute to disease development and progression; (**B**) Diagram showing the transcripts encoded in the 9p21 locus. ANRIL is represented in the diagram as the longest variant reported (CDKN2BAS-1), but several other alternative isoforms have been described.

It seems clear at this point that ANRIL is involved in at least three different mechanisms of epigenetic regulation: initiation of long term repression of CDKN2B locus by complexing SUZ12 in the polycomb repressive complex 2 (PRC2) [25], maintenance of chromatin silencing of the CDKN2A/B locus through interaction with CBX7 in the Polycomb repressive complex 1 (PRC1) [26], and also has been shown to alter DNA methylation of the locus in differentiated cells [61]. However, these functions are apparently not universal, but instead dependent on cell type and sensitive to physiological changes of the cell, such as senescence or differentiation. Our group has reported some effects of ANRIL over CDKN2A expression [21] and other atherosclerosis-related genes [63] in vascular smooth muscle cells (VSMC) that are not explained by the polycomb-repression mechanism. It is not clear whether these regulatory effects are mediated by other chromatin-modifying proteins, by the ANRIL circular isoforms, DNA methylation or other novel mechanism (Figure 1).

Both Kotake *et al.* [25] and Yap *et al.* [26] presented strong experimental evidence demonstrating a regulatory role of ANRIL upon CDKN2A/B through polycomb proteins; however, the role of these polycomb proteins exceeds the CDKN2A/B locus, and they have a well-established participation in remodeling global expression patterns [77,78]. Furthermore, metastasis-associated lncRNA, Hotair, which also associates with PRC2, is involved in the recruitment of SUZ12 and EHZ2 to more than 800 genes [38]. Thus, it would not come as a surprise if ANRIL also is involved in the regulation of several distant loci. The effect of ANRIL splicing variants upon genome-wide chromatin modification changes has not been investigated. However, there is already evidence suggesting that ANRIL has effects over loci other than CDKN2A/B [49,63].

In the light of the new findings regarding ANRIL function, it is appealing to hypothesize that the 9p21 association might be explained by ANRIL-driven regulation of CDKN2A/B and the consequent changes in cellular proliferation. Unquestionably, there is an altered cellular proliferation component in almost all the diseases associated with the locus; however further investigation is required to clearly unravel the role of these genes in the development of these pathologies.

CDKN2A/B locus is certainly associated with cancerogenesis but the connection of this locus with atherosclerosis is less clear. A recent report unveiled a pro-atherogenic role of CDKN2A *in vivo* in mice and a more significant protective role of MTAP (a more distant neighbor of the 9p21 regulatory region) [79]. The relevance of MTAP and the MTAP-ANRIL fusion transcript needs to be investigated to clarify their role in atherogenesis.

Another issue that remains to be clarified is the function of the circular isoforms of ANRIL. A natural antisense transcript forming a circular structure has been shown to be target of nuclear microRNAs and appear to participate in a recently discovered regulatory mechanism in which the circular isoform stabilizes the sense transcript rather than represses it [35]. The possibility of ANRIL circular variants playing a similar role in gene regulation is intriguing, and is definitely worth further analysis.

Clearly, there are several points that remain elusive regarding ANRIL function and its connection to the diseases associated with the 9p21 locus. It is clear that the regulatory mechanisms operating in the region are tissue specific, however, the effects of the polymorphisms over ANRIL expression and the regulatory mechanisms of ANRIL have only been evaluated in few tissues. Determining cell-type specific effects in tissues relevant to atherosclerosis, diabetes and other conditions associated with the locus requires further investigation. The function of ANRIL activating polycomb proteins and repressing the CDKN2A/B locus has been elegantly demonstrated [25,26]; however, its role in global histone remodeling has not been addressed. Finally, ANRIL forms several hair-pin structures along its exons, but the properties of the different regions of the ANRIL transcript and their affinity to bind polycomb proteins has remained unexplored.

All this evidence supports the idea that ANRIL is a key regulatory molecule mediating human disease at different levels and cellular settings. ANRIL is a probable mediator of the Chr9p21 associations and a particularly interesting target for new therapies for a wide range of human diseases.

## Figures and Tables

**Table 1:** Disease-associated SNPs correlated with ANRIL expression.

SNP	Disease association	Reported effect on ANRIL expression
rs564398-A	Risk allele for diabetes and atherosclerotic stroke [65]	Reduced ANRIL expression (exons 1–2). Twice reported to exert the strongest influence in ANRIL expression in peripheral blood [21,23]
rs1063192-C	Risk for glioma [16] and open angle glaucoma [6]	Increased ANRIL expression (exons 1–2) [21,23]
rs1011970-T	Melanoma [13]	Reduced expression of ANRIL (exons 1–2) [23]
rs2151280-T	Risk allele for plexiform neurofibroma development [64] and protective allele for BCC [11]	Reduced expression of ANRIL (exons 15–16) [64]
rs3731217-G	Risk allele for Acute lymphoblastic leukemia [9]	Reduced expression of ANRIL (exons 17–18) [21]
rs496892-G	Risk for atherosclerotic stroke [65] and periodontitis	Reduced ANRIL expression (exons 1–2) [21,23]
rs10757278-G	Lead SNP for CAD risk [66]	Increased ANRIL variant EU741058 expression (exons 1–5 of the long transcript) [22], but reduced expression of ANRIL exons 1–2 [23].
rs3731257-G	Risk allele for ovarian cancer [15]	Increased expression of ANRIL (exons 1–2) [23]
rs10811661-T	Risk allele for diabetes [2]	Reduced ANRIL expression (exons 1–2) [23]

Exon numbers are based on the 19 exon transcript.
